# Diversity of *Acinetobacter baumannii* in Four French Military Hospitals, as Assessed by Multiple Locus Variable Number of Tandem Repeats Analysis

**DOI:** 10.1371/journal.pone.0044597

**Published:** 2012-09-12

**Authors:** Yolande Hauck, Charles Soler, Patrick Jault, Audrey Mérens, Patrick Gérome, Christine Mac Nab, François Trueba, Laurent Bargues, Hoang Vu Thien, Gilles Vergnaud, Christine Pourcel

**Affiliations:** 1 Univ Paris-Sud, Institut de Génétique et Microbiologie, UMR8621, Orsay, France; 2 CNRS, Orsay, France; 3 Laboratoire de biologie clinique, HIA Percy, Clamart, France; 4 Laboratoire de biologie clinique, HIA Bégin, Saint Mandé, France; 5 Service de biologie médicale, HIA Desgenettes, Lyon, France; 6 Service de biologie médicale, HIA Val de Grâce, Paris, France; 7 Bactériologie, Hôpital Armand Trousseau, Assistance Publique-Hôpitaux de Paris (APHP), Paris, France; 8 DGA/MRIS- Mission pour la Recherche et l’Innovation Scientifique, Bagneux, France; University of Iowa Carver College of Medicine, United States of America

## Abstract

**Background:**

Infections by *A. calcoaceticus*-*A. baumannii* (ACB) complex isolates represent a serious threat for wounded and burn patients. Three international multidrug-resistant (MDR) clones (EU clone I-III) are responsible for a large proportion of nosocomial infections with *A. baumannii* but other emerging strains with high epidemic potential also occur.

**Methodology/Principal Findings:**

We automatized a Multiple locus variable number of tandem repeats (VNTR) analysis (MLVA) protocol and used it to investigate the genetic diversity of 136 ACB isolates from four military hospitals and one childrens hospital. *Acinetobacter* sp other than *baumannii* isolates represented 22.6% (31/137) with a majority being *A. pittii*. The genotyping protocol designed for *A.baumannii* was also efficient to cluster *A. pittii* isolates. Fifty-five percent of *A. baumannii* isolates belonged to the two international clones I and II, and we identified new clones which members were found in the different hospitals. Analysis of two CRISPR-cas systems helped define two clonal complexes and provided phylogenetic information to help trace back their emergence.

**Conclusions/Significance:**

The increasing occurrence of *A. baumannii* infections in the hospital calls for measures to rapidly characterize the isolates and identify emerging clones. The automatized MLVA protocol can be the instrument for such surveys. In addition, the investigation of CRISPR/cas systems may give important keys to understand the evolution of some highly successful clonal complexes.

## Introduction

The genus *Acinetobacter* currently counts 27 validly named species (http://www.bacterio.cict.fr/a/acinetobacter.html) and several unnamed provisional species and genomic species [1], [2]. *Acinetobacter baumannii*, *Acinetobacter pittii* sp. nov (formally *Acinetobacter* gen. sp. 3) and *Acinetobacter nosocomialis* sp. nov (formally *Acinetobacter* gen. sp. 13 TU) [3] are mostly associated with clinical issues [4]. These species and the closely related species *A. calcoaceticus*, an environmental species, are difficult to distinguish phenotypically, which has led to the proposal to lump them together into the *A. calcoaceticus*-*A. baumannii* (ACB) complex [3], [5]. *A. baumannii* infection has almost exclusively been observed in critically ill hospitalized patients [6]. The emergence of resistance to multiple antibiotics including carbapenems in *A. baumannii* is worrying [7], [8], [9]. Three clonal lineages (European (EU) clones I–III) of multidrug resistant (MDR) and extensively-drug resistant (XDR) *A. baumannii* strains have been found to be associated with outbreaks in hospitals at different locations [10], [11]. EU clones I and II are now known to occur worldwide [8]. Antimicrobial resistance and epidemic spread of strains of *A. pittii* and *A. nosocomialis* is still limited compared to *A. baumannii* [12], [13].

The natural reservoir of *A. baumannii* clinical isolates is unknown although *A. baumannii* was isolated from soil, meat, fish and vegetables [14], [15]. Different *Acinetobacter* species including *A. baumannii* were found to grow in the rhizosphere of wheat [16]. The coexistence of different *Acinetobacter* species in a single environment might favor genomic transfer, a major source of genetic diversity [17], [18]. The implication of *A. baumannii* in combat casualties was investigated in several studies, as these patients often suffer from severe burns and wounds and are initially treated in field hospitals [19], [20]. In a recent study, 65 isolates from 48 patients were investigated showing the presence of European clones I to III genotypes and of other emerging genotypes defined by amplified fragment length polymorphism (AFLP) typing [19]. The difficulty in evaluating the genetic diversity of isolates infecting these patients worldwide is the use of different genotyping techniques by different laboratories. AFLP and other pattern-based genotyping methods such as pulse field gel electrophoresis (PFGE) [21] or rep-PCR typing [22] are currently used but the results cannot easily be compared between laboratories or stored into databases. By contrast, Multilocus sequence typing (MLST) schemes [23], [24], [25], [26] and multiple locus variable number of tandem repeats (VNTR) analysis (MLVA) schemes [27], [28], [29] provide a genotype in the form of a code that can be easily shared. Both approaches allow the identification of clonal lineages and the investigation of population structure [26], [30], [31]. In addition, one proposed MLVA-8 scheme was capable of high resolution sub-typing in a study of international clonal lineage II [32].

Clustered Regularly Interspersed (CRISPR) elements are DNA structures made of short repeats separated by stretches of DNA called spacers derived from invading nucleic acids and which number can vary from one strain to another [33]. Together with genes called *cas* for “CRISPR –associated”, they constitute a widespread adaptive immune system in prokaryotes [34]. They appear to be transferable in an intra and interspecies manner [35]. CRISPR polymorphism has been used to genotype strains and to perform phylogenetic analyses in a number of bacterial species [36]. Two CRISPR-cas systems have been found in the genome of several *A. baumannii* strains and in the *A. bayli* ADP1 strain [37].

In the present investigation we automatized the previously published MLVA-8 scheme and applied it to the typing of a collection of ACB complex isolates from four military and one civilian hospitals. Presence of the CRISPR-cas systems was also investigated to evaluate their distribution and further define some clones.

## Results

### Automatization of the MLVA Assay

We previously described the identification of 10 VNTRs in the genome of three *A. baumannii* strains available at that time, and the selection of 8 of those for an efficient MLVA-8 genotyping scheme (which will be called MLVA-8_Orsay_ to unambiguously identify this selection of 8 loci). The protocol involved individual PCR amplifications and agarose gel electrophoresis [29]. In the present study we included the 10 VNTRs (MLVA-10_Orsay_) and automatized the assay in order to accelerate the procedure and to obtain a more accurate measurement of alleles for the 5 microsatellites loci. The VNTRs were amplified in two multiplex PCRs (Table 1) and the products were analysed on a capillary electrophoresis device. The conversion of amplicon size to repeat number was performed automatically by the CEQ8000 Genetic Analysis System software. The new protocol was first successfully tested on six reference strains (ATCC 17978, ACICU, AYE, RUH 134, RUH 875, RUH 5875 listed in Table S1) previously typed manually using agarose gel electrophoresis, showing identical results (the values are given in Table S2). In order to test whether MLVA-10_ Orsay_ could be used for typing the different members of the ACB complex, we genotyped five *A. pittii* and five *A. nosocomialis* reference strains (Table S2). Polymorphism was observed in the different samples and at least 8 out of 10 VNTRs could be amplified. Three loci, Abaum3002, Abaum3530 and in particular Abaum0826 were responsible for amplification failures. VNTR amplification was not observed when DNA of species not belonging to the ACB complex was analysed.

**Table 1:** Oligonucleotides used for the multiplex PCR reactions.

	VNTRa	Oligonucleotide	TM °C	Repeatsize (bp)	Size of flanking regions (bp)^b^	Allele range (bp)^c^	
**Multiplexe 1**	Abaum_3406_L_D4	CACTATATTGAAGTGCTTTTA	48	30	231	342	669
	Abaum_3406_R	GTGGTTTTTCTATTGGTACATTAC	58				
	Abaum_826_L_D3	TGACTACTGAAACAGTTTTTG	56	9	361	415	649
	Abaum_826b-R	ACTTGGTTTGAGCTATAGAA	54				
	Abaum_17_L_D3	GTGAGGGTAGAGTATTTGCTC	62	9	80	85	346
	Abaum_17_R	GAGTTAGGGAGTCTTTTATATGG	64				
	Abaum_845b_L_D2	CCAAATTGCTCCAATATCTGAACT	66	7	115	129	346
	Abaum_845b_R	GCAAATTTATACAGCTCAAGAAAATGG	72				
	Abaum_2396_L_D4	CAAGTCCAATCAACTCATGATG	62	6	105	147	315
	Abaum_2396_R	CTCCTGTAAGTGCTGTTCAGCC	68				
	Abaum_3468_Lb_D2	AAGACCACTTTGCAACTAAA	58	6	308	350	429
	Abaum_3468_Rb	TTTGCTGATGGTTGTGGCTA	58				
**Multiplexe 2**	Abaum _3530_L_D2	TGCAACCGGTATTCTAGGAAC	62	60	121	162	642
	Abaum _3530_R	CCTTGAACAACATCGATTACTGGA	66				
	Abaum_3002_L_D3	GACTGAAGCAAGACTAAAACGT	62	57	121	428	596
	Abaum_3002_R	TCTGGGCAGCTTCTTCTTGAGC	68				
	Abaum_2240b_L_D4	AAGGTTTCCGTGTTGAACGTGATT	68	99	209	204	538
	Abaum_2240b_R	CAGGTGTCAAATCGTAAACAAA	60				
	Abaum_1988_L_D3	GGCAAGGCATGCTCAAGGGCC	70	26	77	136	447
	Abaum_1988_R	CAGTAGACTGCTGGTTAATGAG	64				

a The forward primer is labeled with one of three dyes D2, D3 and D4. The allele range for each reaction is indicated showing that amplicons with the same dye cannot overlap.

b Values referred to the genome of strain ATCC 17978 and considered for allele assignment.

c The allele range for each reaction is indicated showing that amplicons with the same dye cannot overlap. Values obtained with *A. baumannii* isolates.

### Investigation of ACB Complex Isolates in Five French Hospitals

**Figure 1:**
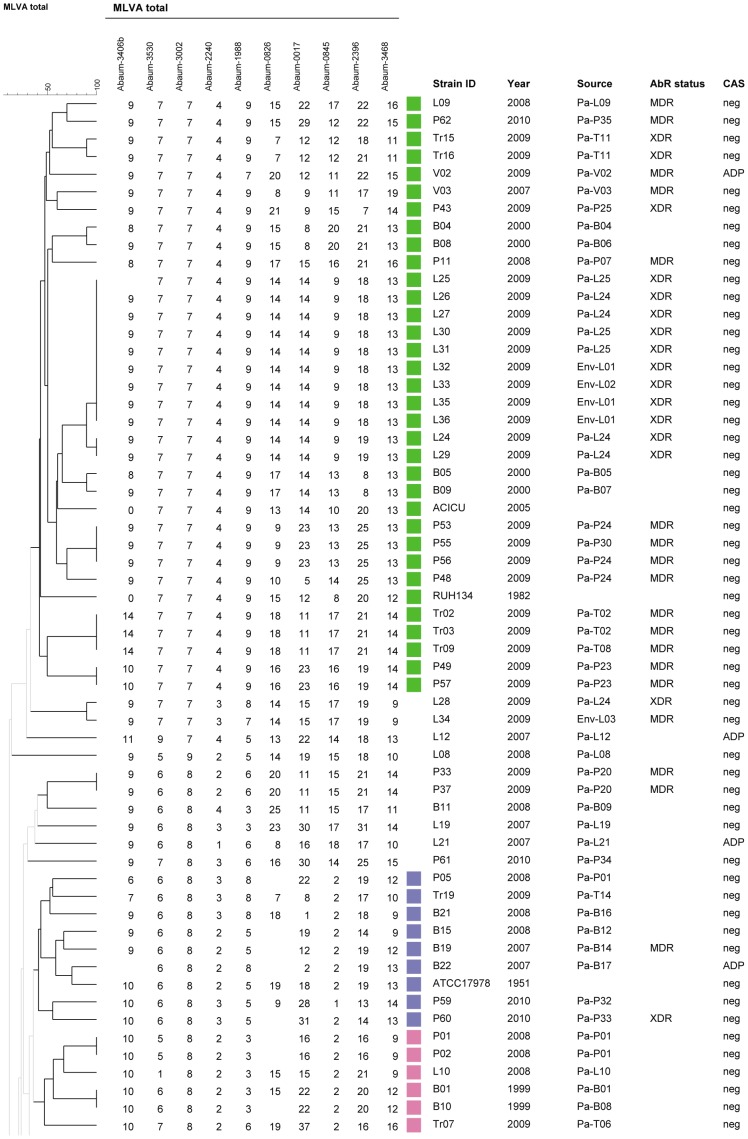
Genetic diversity of 59 *A. baumannii* isolates. A 40% cut-off value was used to define MLVA clusters, shown with different colors when containing 5 isolates or more. On the side are indicated the year of isolation, the source, the antibiotics resistance status and the existence of a CRISPR -cas system.

**Figure 2:**
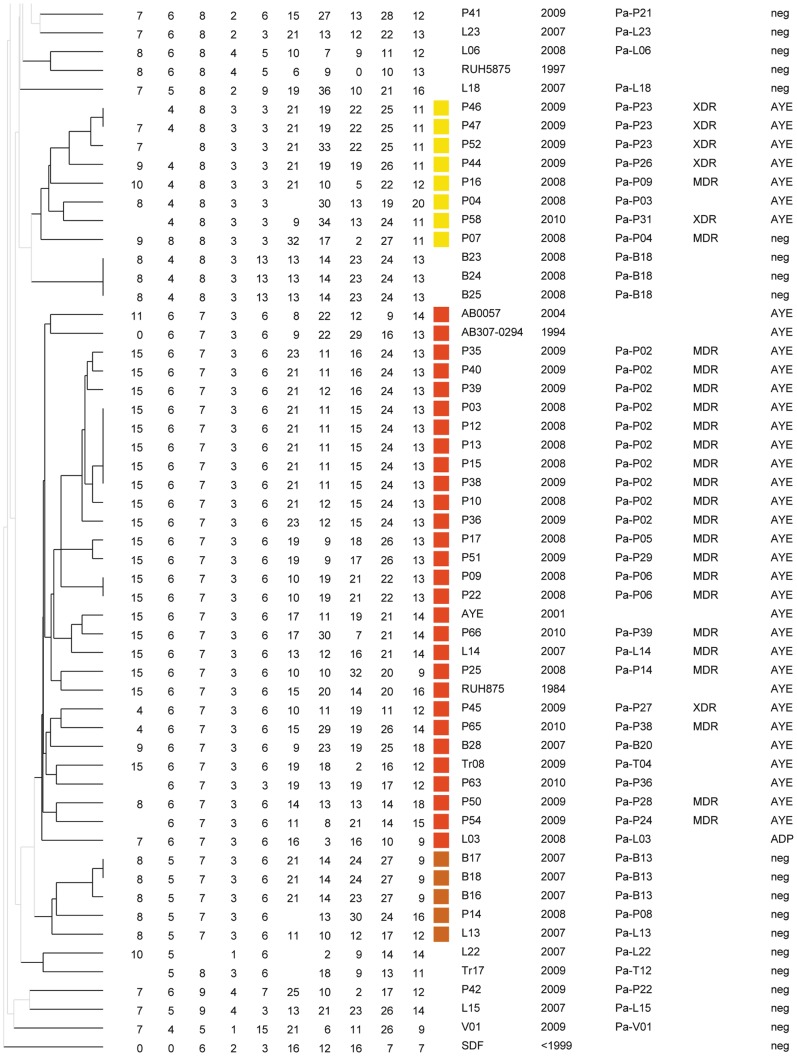
Genetic diversity of 56 *A. baumannii* isolates. The legend is that of Figure 1.

We wished to investigate the diversity of isolates recovered from military hospitals which mostly care for wounded and burn patients, soldiers and civilians (Table S1). We also included in the analysis twelve isolates from the A. Trousseau hospital in Paris which cares for children with different pathologies. Among 136 ACB complex isolates, *rpoB* sequencing identified 27 *A. pittii* (genomic species 3) isolates, 2 *A. nosocomialis* isolates (genomic species 13TU), 1 *Acinetobacter* sp isolate (98% identity with *A. oleivorans*) and 106 *A. baumannii* isolates. The calculated typeability of MLVA-10_Orsay_ on *A. baumannii* samples was 98%, as missing values occurred for Abaum0826, Abaum3406 and Abaum3002. The absence of amplification could be due to mismatches in the target region of primers or to complete or partial deletion of the locus, but this was not confirmed. Therefore absent data were not considered as a character in the clustering analysis. Figure 1 and Figure 2 show a clustering analysis of *A. baumannii* isolates only. With a cut-off value of 40% corresponding to the existence of identical size alleles for at least 4 VNTRs, 11 clusters (two isolates or more) and 13 singletons were observed. As shown with green and red colors on Figure 1 and Figure 2, 53% (56/106) of the isolates fell into the two larger clusters respectively including RUH 134 the reference strains for EU clone I (32 isolates) and RUH 875 the reference strain for EU clone II (24 isolates). The majority of isolates in these clusters were multidrug resistant whereas those in smaller clusters, and non-epidemic isolates (singletons), were generally sensitive to several classes of antibiotics. Interestingly 7 out 8 isolates in a small cluster from 6 patients in the Percy hospital (shown in yellow color and called here the Percy cluster) were XDR or MDR. Only one isolate (L06) clustered with RUH 5875, the reference strain for EU clone III. Abaum0826 was not amplified in 13% (15/116) of samples which do not belong to the EU clone I and EU clone II, and our efforts to design new primers that amplify all the isolates failed.

Calculated typeability of MLVA-10_Orsay_ on other members of the ACB complex was 86.5% due to missing data for VNTRs Abaum3002, Abaum3530 and Abaum0826. The dendrogram on Figure S1 reveals the existence of some groups (shown with different colors) sharing 4 VNTR alleles and possibly reflecting an epidemic situation. All isolates except B20 were susceptible to antibiotics.

A clustering analysis was performed with a total of 173 ACB complex isolates including those previously typed by MLVA [29], and the result showed that although most strains clustered according to the species, there was some inter-species clustering as shown on the minimum spanning tree in Figure S2.

### CRISPR Analysis

We wished to investigate the presence of CRISPR-cas systems in members of the ACB complex and evaluate their potential use for epidemiological and phylogenetic studies. We searched by PCR for the presence of the *cas1* gene from the two known CRISPR-cas systems. The first one is present in genome sequence data from *A. baumannii* AYE, AB0057 and AB307 (called here the AYE system), and the second is found in *A. baylii* ADP1 and in the *A. baumannii* type strain ATCC 19606^T^ (called here the ADP system). The AYE *cas1* gene was present in all the EU clone I isolates as defined by MLVA-10_Orsay_ except in isolate L03 placed in an outgroup position (Figure 2). All the isolates in the Percy cluster possessed an AYE *cas1* gene. The sequence of the A28 (EU clone I) and P16 (Percy cluster) *cas1* amplicons was identical to that of the AYE strain. The ADP *cas1* gene was found in four isolates dispersed throughout the tree; A22, L21, V02 and L03 (Figures 1 and 2).

**Figure 3:**
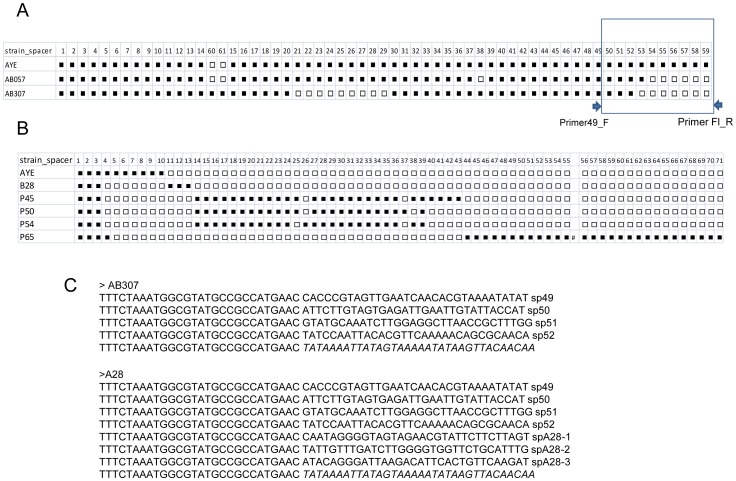
Polymorphism of the AYE CRISPR locus in *A. baumannii*. A) Schematic representation of the CRISPR locus in three sequenced genomes and at the growing end of isolates A28, P054 and P065. Arrows show the position of primers used to amplify a portion of the locus. Specific spacers are shown with grey boxes. B) Sequence of the growing end of strain AB307 and isolate A28. The sequence flanking the last DR is in italics.

We then studied whether the AYE CRISPR was polymorphic, ie. whether the spacer composition was different in the different isolates. The number of spacers in the CRISPR locus of strains AYE, AB057 and AB307 are respectively 59 (3569bp long), 52 (3149 bp long) and 45 (2729 bp long) (Figure 3A). They share the majority of their spacers except those present at the growing end, as initially described in *Yersinia pestis* [38]. To facilitate the analysis of the CRISPR polymorphism in the different isolates, we amplified only the portion where new spacers are added. The PCR forward primer was selected inside spacer 49 of strain AYE as shown on Figure 3A and the reverse primer inside the sequence flanking the last motif. Amplification of strains AYE, AB0057 and AB307 were expected to provide amplicons of respectively 729 bp, 369 bp and 309 bp according to sequenced genome data, and this was confirmed experimentally (Figure S3 and data not shown). We then tested all the isolates possessing the AYE *cas* 1 gene and others closely related by genotyping (part of the result is shown in Figure S3). Isolates in Percy cluster all produced a 309 bp fragment but the amplification was weak suggesting that the primers did not match perfectly. The majority of isolates in the EU clone I produced a 369 bp amplicon, and interestingly larger amplicons were observed for isolates placed in an outgroup position: P50, P54, P45, P65 and A28 produced fragments of respectively 1700 bp, 1700 bp, 1800 bp, 2300 bp and 490 bp. Complete (A28) or partial sequencing of these amplicons revealed the presence of new unique spacers, some of them matching chromosomal sequences or plasmids (Figure 3B and Table S3).

## Discussion

### MLVA

We describe an improved automatised MLVA assay which allows the rapid and efficient genotyping of *A. baumannii* isolates and may also be helpful to genotype isolates of other members of the ACB complex. MLVA-10_Orsay_ allows the definition of clusters of isolates, some of which correspond to known clonal lineages and others apparently emerging locally. The four minisatellites also present in the MLVA-8_Orsay_ scheme (Abaum3530, Abaum3002, Abaum2240, Abaum1988) are very informative to distribute isolates into majors clones, such as EU clone I and EU clone II. Inside these clones polymorphism is mostly provided by the fifth minisatellite, Abaum3406 and by the 5 microsatellites (Abaum0826, Abaum0017, Abaum0845, Abaum2396, Abaum3468) which bring a high level of discrimination. Abaum3406, a 30 bp repeat unit VNTR, is very polymorphic even inside lineages, as compared to the four other large-repeat units VNTRs. It encodes a portion of the copper resistance protein A precursor (also called multicopper oxydase), characterized by the presence of repeated amino-acids, with a high degree of variability. Copper-based biocids are used to fight health-care associated organisms and might exert a selective pressure for resistance [39]. The possible role of repeat number variation in the gene bearing Abaum3406 needs to be further investigated.

### Persistence and Evolution of MDR Strains

The MDR and XDR isolates are mostly distributed in three MLVA clones, two of which correspond to EU clones I and II and the third being constituted of 7 isolates from 6 patients of Percy hospital. The definition of this local clone is further sustained by the presence of the AYE CRISPR-cas system in the 7 isolates (see dedicated paragraph below). Two other clones with mostly antibiotics-susceptible isolates from different hospitals were observed, one of which include the ATCC 17978 strains. The distribution of isolates from Trousseau hospital was similar to that of the military hospitals, although their small number preclude statistical analyses. It would be interesting to genotype by MLVA isolates from additional reference clones previously analysed by different genotyping techniques in order to better compare the bacterial populations [26]. This will be the subject of future collaborative studies.

Multiple isolates were recovered from 12 patients (2 to 10) and in 5 of them they showed the same genotype. In the other patients, variants of the same genotype were observed, differing at one or two small-size-repeat VNTRs, and in addition, 4 patients (P01, P23, P24, L24) harbored isolates of unrelated genotypes. In the Percy cluster, the first isolate recovered in 2008 was susceptible to all antibiotics except cotrimoxazol (SXT). Isolates of the same clonal lineage were found in another patient later in 2008, then in 2009 and 2010 and they were all MDR or XDR. The patients were in different wards. These observations suggest that a complex population of variants of this clone persists in the hospital. Some patients stay for long periods of time in the hospital and may be reservoir for *A. baumannii*. During a period of one year, 10 EU clone I isolates were recovered from burn patient Pa-P02 with 6 genotypes due to differences at VNTRs Abaum0826, Abaum0017 and Abaum0845. Although they were always susceptible to at least two classes of antibiotics these strains could not be eliminated. In 2009 two patients from hospital Desgenettes were infected with a XDR strain of EU clone II (Pa-L24 and Pa-L25) and the strain could be found on different environmental sources, including a door handle and a bench (Env-L01 and Env-L02). Over a period of three months, 3 genotype variants could be observed. It is possible that different genotypes coexist in a single source and in the future it will be interesting to systematically analyze several isolates from the same culture to test this hypothesis. The automated MLVA method will make such investigations easier.

Some of the military patients were wounded in other countries and were first treated in field hospitals before being transferred to France. We did not find any relationship between the isolates’ genotype and the localization of the field hospital. Therefore we have no indication on the source of infection.

### Clinical Outcome

*A. pittii* isolates represent 20% of the *Acinetobacter* isolates and a third of them form a cluster. This observation is interesting because it suggests the existence of clonal lineages spreading to different locations, similarly to what is observed with *A. baumannii*. These strains are susceptible to antibiotics except fosfomycin as previously reported [40], [41]. They were found mostly in blood from patients having a deep vein catheter whereas infections of wounds and skin were mostly due to *A. baumannii*. Difference in clinical outcome depending on the genome species has been previously investigated [42], showing that *A. baumannii* was more frequently associated with pneumonia. Wisplinghoff et al. investigated 295 isolates from patients in the US with bloodstream infection and found that one third was due to *A. nosocomialis* and *A. pittii* [43]. In other studies *A. pittii* were found at similar or even higher levels than *A. baumannii*, independently of the source [12], [44]. In the present work and in others, *A. baumannii* was less susceptible to antibiotics than *A. nosocomialis* and *A. pittii* [45], [46].

### The CRISPR-cas Systems

We searched for the presence of the CRISPR-cas systems observed in some sequenced genomes to evaluate their potential for genotyping and to help in defining clonal complexes. The majority of isolates do not possess either one of the described systems which prevent their use as a tool for genotyping. However, the AYE CRISPR-cas system was very informative to investigate the diversity of the EU clone I lineage. Five isolates possess longer CRISPRs and this may suggest that they have further evolved in the environment as compared to the rest of the clone. Isolate L03 placed in an outgroup position, does not possess the AYE CRISPR-cas system and may represent an ancestral genotype for this clone. All isolates in the Percy cluster possess an EU clone I CRISPR-cas system indicating that they may be phylogenetically related to this clone but this requires further investigation by whole genome draft sequencing analysis. The five isolates with the ADP CRISPR-cas system are distributed in different clusters suggesting that the system may have been acquired by horizontal transfer at different time during evolution. Therefore we believe that investigation of these elements, although restricted to a subpopulation of isolates, may provide interesting phylogenetic information to understand the emergence of major lineages.

## Materials and Methods

### Ethics Statement

Strains were collected from different specimens as part of the patients’ usual care, without any additional sampling. A code was used to refer to each patient without offering any possibility to trace these patients, and no information was reported except the sample source. Ethic committees in each hospital providing bacterial isolates were consulted and they declared that patient informed consent was not needed: for the military hospitals the “Comité d’éthique et des experimentations” and for hospital Armand Trousseau the “Comité Consultatif pour la Protection des Personnes dans la Recherche Biomédicale (CCPPRB) Ile-De-France – Paris – Saint Antoine”.

### Bacterial Samples

The isolates were obtained from specimens of patients from different wards or from hospital environment (Table S1). A total of 124 ACB complex isolates were collected in four military hospitals (from soldiers and civilian patients) from 2007 to 2010: 60 were from Hopital d’Instruction des Armées (HIA) Percy, 35 isolates from HIA Desgenettes, 6 from HIA du Val de Grâce, 23 from HIA Begin. In addition 12 ACB complex isolates were collected from Armand Trousseau children hospital in Paris. In some cases several isolates were recovered from a single patient (up to ten for patient Pa-P02). Samples were streaked across Mueller-Hinton agar and incubated at 37°C for 24 hours. Bacteria were identified as belonging to ACB complex using standard automated biochemical testing methods. *Acinetobacter baumannii* were tested using API 20 NE strip or API 20 E (bioMerieux, Marcy l’Etoile) and *Acinetobacter spp* with API 20 NE. To differentiate between the different members of the ACB complex a portion of the *rpoB* gene which is highly variable and shows species specific characteristics was amplified and sequenced (see below). Clinical isolates from other related species, recovered in Percy and Trousseau hospitals, were included to check the specificity of the assay: *A. lwofii* (6 isolates), *A. ursingii, A. haemolyticus, A. junii, Acinetobacter sp*. (4 isolates), *Enterobacter cloacae, P. aeruginosa*.

DNA samples from five *A. nosocomialis* isolates and five *A. pittii* isolates were generously provided by L. Dijkshoorn, Leiden University Medical Center. DNAs from ATCC 17978, AYE (EU clone I) and ACICU (EU clone II) strains were generously provided by Paolo Visca (University Roma Tre).

### DNA Purification and VNTR Analysis

Genomic DNA was extracted using the QIAamp DNA Mini Kit (Quiagen SAS-France, Courtaboeuf). Alternatively thermolysates were prepared by boiling bacteria in water for 20 minutes. The VNTRs were those described in [29]. Five were of the minisatellite class with 9 bp or longer repeats (Abaum3406, Abaum3530, Abaum3002, Abaum2240, Abaum1988) and 5 of the microsatellite class with smaller than 9 bp repeats (Abaum0826, Abaum0017, Abaum0845, Abaum2396, Abaum3468). The 10 VNTRs were amplified in two multiplex PCRs and using the Qiagen multiplex kit as recommended by the supplier (Table 1). The forward primer was labeled with one of three Well-Red dyes, D2, D3 or D4 (Sigma-Aldrich, St Quentin Fallavier, France). PCR primers were redesigned for four VNTRs so that allele ranges do not overlap (Abaum0826, Abaum2240 and Abaum3468) or to improve the efficiency of PCR amplification (Abaum0845). Capillary gel electrophoresis was performed on a CEQ8000 automatic DNA Analyser (Beckman-Coulter, USA) as described [47]. VNTR profiles of reference strains RUH 134 (EU clone I), RUH 875 (EU clone II), RUH 5875 (EU clone III), previously genotyped by MLVA were used for comparison [29].

### PCR Amplification and Sequencing

For species identification, a 350-bp fragment of the *rpoB* gene hypervariable zone 1 was amplified using primers Ac696F and Ac1093R [48]. Sequencing was performed from one end using the Ac696F PCR primer (Beckman Coulter Genomics, UK) and new sequence data were deposited in GenBank. The primers used to detect the AYE *cas1* gene were Abaum-Cas1-F 5′ TCAAGCTGCGATGCGAATGT3′ and Abaum-Cas1-R 5′ ATCCGGGCAAATTGAAACGC3′ giving rise to a 210 bp fragment. The primers used to detect the *A. bayli* ADP1 *cas1* gene were Cas1-ADP_F 5′AAGCATTTTCATCAATATAAATAC3′ and Cas1-ADP_R 5′TTATAATATTCCAGAGAAAAACA3′ giving rise to a 344 bp fragment.

CRISPR_AYE_49_F 5′ CCCGTAGTTGAATCAACACGTA 3′ and CRISPR_AYE_Fl_R 5′ TTTGATTGGGTAAAATGCCAAA3′were used to amplify one end of the AYE CRISPR locus. Sequencing was performed from both ends using the PCR primers (Beckman Coulter Genomics, UK). All new sequence data has been deposited in GenBank under the accession numbers HE801220 to HE801224 and HE803768 to HE803825.

Complete genome sequences used in this study were: *A. baumannii* strains AB0057 (GenBank:http://www.ncbi.nlm.nih.gov.gate1.inist.fr/nucleotide/213155370/CP001182-3) and AB307 (Genbank: CP001172.1) [49], ACICU (GenBank: NC_010611) [50], ATCC17978 (GenBank: NC_009085) [51], AYE (GenBank: NC_010410), SDF (Genbank: CU468230.2) [52]) and ADP1 (Genbank CR543861) [53]. The sequence of *A. baumannii* ATCC 19606^T^ was recovered from the Acinetobacter group sequencing project, Broad Institute of Harvard and MIT (http://www.broadinstitute.org/).

## Supporting Information

Figure S1 **Genetic diversity of 40 non-** ***A. baumannii*** **ACB complex isolates.** A 40% cut-off value was used to define MLVA clusters, shown with different colors when containing 3 isolates or more. On the side are indicated the year of isolation, the patient code (source), the site of isolation (removal) and the Acinetobacter species.
(TIF)

Figure S2 **Minimum spanning analysis of MLVA data for 173 ACB complex isolates.** Isolates from the present study belonging to the two larger *A. baumannii* clonal complexes and to the *pittii* and *nosocomialis* species are shown with colors.
(TIF)

Figure S3 **Polymorphism of the AYE CRISPR locus.** Agarose gel electrophoresis of amplicons corresponding to the CRISPR growing end. The analysed samples are A) P03, P04, P09, P12, P15, P16, P17, P22, P25, P35, P36, P39, P40, L04, AYE, A28, R12, R17, P44, P07, P46, P47. B) P66, P65, P63, P54, P14, Tr08, P51, P50.
(TIF)

Table S1 **List of isolates used in the present study.** An initial is used for the different hospitals: P Percy, L Lyon, V Val de Grâce, B Béjin, Tr Trousseau.
(DOC)

Table S2 **Result of MLVA genotyping of reference strains.**
(DOCX)

Table S3 **List of spacers identified in sequenced AYE CRISPR loci.** ^1^ The table corresponds to the dictionary produced by CRISPRcompar [Grissa, 2008 #893] where spacers are annotated in each analysed sample as shown in the second column.
(DOCX)

## References

[1] Dijkshoorn L, Nemec A, Seifert H (2007) An increasing threat in hospitals: multidrug-resistant *Acinetobacter baumannii* . Nat Rev Microbiol 5: 939–951. 18007677 10.1038/nrmicro1789

[2] Nemec A, De Baere T, Tjernberg I, Vaneechoutte M, van der Reijden TJ, et al (2001) *Acinetobacter ursingii* sp. nov. and *Acinetobacter schindleri* sp. nov., isolated from human clinical specimens. Int J Syst Evol Microbiol 51: 1891–1899. 11594623 10.1099/00207713-51-5-1891

[3] Nemec A, Krizova L, Maixnerova M, van der Reijden TJ, Deschaght P, et al (2011) Genotypic and phenotypic characterization of the *Acinetobacter calcoaceticus-Acinetobacter baumannii* complex with the proposal of *Acinetobacter pittii* sp. nov. (formerly Acinetobacter genomic species 3) and *Acinetobacter nosocomialis* sp. nov. (formerly Acinetobacter genomic species 13TU). Res Microbiol 162: 393–404. 21320596 10.1016/j.resmic.2011.02.006

[4] Peleg AY, Seifert H, Paterson DL (2008) *Acinetobacter baumannii*: emergence of a successful pathogen. Clin Microbiol Rev 21: 538–582. 18625687 10.1128/CMR.00058-07 PMC2493088

[5] Gerner-Smidt P, Tjernberg I, Ursing J (1991) Reliability of phenotypic tests for identification of *Acinetobacter* species. J Clin Microbiol 29: 277–282. 2007635 10.1128/jcm.29.2.277-282.1991 PMC269753

[6] Fournier PE, Richet H (2006) The epidemiology and control of *Acinetobacter baumannii* in health care facilities. Clin Infect Dis 42: 692–699. 16447117 10.1086/500202

[7] Perez F, Hujer AM, Hujer KM, Decker BK, Rather PN, et al (2007) Global challenge of multidrug-resistant *Acinetobacter baumannii* . Antimicrob Agents Chemother 51: 3471–3484. 17646423 10.1128/AAC.01464-06 PMC2043292

[8] Higgins PG, Dammhayn C, Hackel M, Seifert H (2010) Global spread of carbapenem-resistant *Acinetobacter baumannii* . J Antimicrob Chemother 65: 233–238. 19996144 10.1093/jac/dkp428

[9] Magiorakos AP, Srinivasan A, Carey RB, Carmeli Y, Falagas ME, et al.. (2011) Multidrug-resistant, extensively drug-resistant and pandrug-resistant bacteria: an international expert proposal for interim standard definitions for acquired resistance. Clin Microbiol Infect. 10.1111/j.1469-0691.2011.03570.x 21793988

[10] Dijkshoorn L, Aucken H, Gerner-Smidt P, Janssen P, Kaufmann ME, et al (1996) Comparison of outbreak and nonoutbreak *Acinetobacter baumannii* strains by genotypic and phenotypic methods. J Clin Microbiol 34: 1519–1525. 8735109 10.1128/jcm.34.6.1519-1525.1996 PMC229053

[11] van Dessel H, Dijkshoorn L, van der Reijden T, Bakker N, Paauw A, et al (2004) Identification of a new geographically widespread multiresistant *Acinetobacter baumannii* clone from European hospitals. Res Microbiol 155: 105–112. 14990262 10.1016/j.resmic.2003.10.003

[12] van den Broek PJ, van der Reijden TJ, van Strijen E, Helmig-Schurter AV, Bernards AT, et al (2009) Endemic and epidemic acinetobacter species in a university hospital: an 8-year survey. J Clin Microbiol 47: 3593–3599. 19794057 10.1128/JCM.00967-09 PMC2772585

[13] Lee YC, Huang YT, Tan CK, Kuo YW, Liao CH, et al (2011) *Acinetobacter baumannii* and *Acinetobacter* genospecies 13TU and 3 bacteraemia: comparison of clinical features, prognostic factors and outcomes. J Antimicrob Chemother 66: 1839–1846. 21653602 10.1093/jac/dkr200

[14] Berlau J, Aucken HM, Houang E, Pitt TL (1999) Isolation of Acinetobacter spp. including *A. baumannii* from vegetables: implications for hospital-acquired infections. J Hosp Infect 42: 201–204. 10439992 10.1053/jhin.1999.0602

[15] Houang ET, Chu YW, Leung CM, Chu KY, Berlau J, et al (2001) Epidemiology and infection control implications of *Acinetobacter* spp. in Hong Kong. J Clin Microbiol 39: 228–234. 11136776 10.1128/JCM.39.1.228-234.2001 PMC87707

[16] Sachdev D, Nema P, Dhakephalkar P, Zinjarde S, Chopade B (2010) Assessment of 16S rRNA gene-based phylogenetic diversity and promising plant growth-promoting traits of *Acinetobacter* community from the rhizosphere of wheat. Microbiol Res 165: 627–638. 20116982 10.1016/j.micres.2009.12.002

[17] Fournier PE, Vallenet D, Barbe V, Audic S, Ogata H, et al (2006) Comparative genomics of multidrug resistance in *Acinetobacter baumannii* . PLoS Genet 2: e7. 16415984 10.1371/journal.pgen.0020007 PMC1326220

[18] Snitkin ES, Zelazny AM, Montero CI, Stock F, Mijares L, et al (2011) Genome-wide recombination drives diversification of epidemic strains of *Acinetobacter baumannii* . Proc Natl Acad Sci U S A 108: 13758–13763. 21825119 10.1073/pnas.1104404108 PMC3158218

[19] Petersen K, Cannegieter SC, van der Reijden TJ, van Strijen B, You DM, et al (2011) Diversity and clinical impact of *Acinetobacter baumannii* colonization and infection at a military medical center. J Clin Microbiol 49: 159–166. 21084513 10.1128/JCM.00766-10 PMC3020478

[20] Scott P, Deye G, Srinivasan A, Murray C, Moran K, et al (2007) An outbreak of multidrug-resistant *Acinetobacter baumannii*-*calcoaceticus* complex infection in the US military health care system associated with military operations in Iraq. Clin Infect Dis 44: 1577–1584. 17516401 10.1086/518170

[21] Seifert H, Dolzani L, Bressan R, van der Reijden T, van Strijen B, et al (2005) Standardization and interlaboratory reproducibility assessment of pulsed-field gel electrophoresis-generated fingerprints of *Acinetobacter baumannii* . J Clin Microbiol 43: 4328–4335. 16145073 10.1128/JCM.43.9.4328-4335.2005 PMC1234071

[22] Carretto E, Barbarini D, Dijkshoorn L, van der Reijden TJ, Brisse S, et al (2011) Widespread carbapenem resistant *Acinetobacter baumannii* clones in Italian hospitals revealed by a multicenter study. Infect Genet Evol 11: 1319–1326. 21554997 10.1016/j.meegid.2011.04.024

[23] Bartual SG, Seifert H, Hippler C, Luzon MA, Wisplinghoff H, et al (2005) Development of a multilocus sequence typing scheme for characterization of clinical isolates of *Acinetobacter baumannii* . J Clin Microbiol 43: 4382–4390. 16145081 10.1128/JCM.43.9.4382-4390.2005 PMC1234098

[24] Ecker JA, Massire C, Hall TA, Ranken R, Pennella TT, et al (2006) Identification of *Acinetobacter* species and genotyping of *Acinetobacter baumannii* by multilocus PCR and mass spectrometry. J Clin Microbiol 44: 2921–2932. 16891513 10.1128/JCM.00619-06 PMC1594644

[25] Hamouda A, Evans BA, Towner KJ, Amyes SG (2010) Characterization of epidemiologically unrelated *Acinetobacter baumannii* isolates from four continents by use of multilocus sequence typing, pulsed-field gel electrophoresis, and sequence-based typing of bla(OXA-51-like) genes. J Clin Microbiol 48: 2476–2483. 20421437 10.1128/JCM.02431-09 PMC2897490

[26] Diancourt L, Passet V, Nemec A, Dijkshoorn L, Brisse S (2010) The population structure of *Acinetobacter baumannii*: expanding multiresistant clones from an ancestral susceptible genetic pool. PLoS One 5: e10034. 20383326 10.1371/journal.pone.0010034 PMC2850921

[27] Turton JF, Matos J, Kaufmann ME, Pitt TL (2009) Variable number tandem repeat loci providing discrimination within widespread genotypes of *Acinetobacter baumannii* . Eur J Clin Microbiol Infect Dis 28: 499–507. 19020910 10.1007/s10096-008-0659-3

[28] Turton JF, Baddal B, Perry C (2011) Use of the accessory genome for characterization and typing of *Acinetobacter baumannii* . J Clin Microbiol 49: 1260–1266. 21289143 10.1128/JCM.02335-10 PMC3122835

[29] Pourcel C, Minandri F, Hauck Y, D’Arezzo S, Imperi F, et al (2011) Identification of variable-number tandem-repeat (VNTR) sequences in *Acinetobacter baumannii* and interlaboratory validation of an optimized multiple-locus VNTR analysis typing scheme. J Clin Microbiol 49: 539–548. 21147956 10.1128/JCM.02003-10 PMC3043498

[30] Vergnaud G, Pourcel C (2009) Multiple locus variable number of tandem repeats analysis. Methods Mol Biol 551: 141–158. 19521873 10.1007/978-1-60327-999-4_12

[31] Visca P, D’Arezzo S, Ramisse F, Gelfand Y, Benson G, et al (2011) Investigation of the population structure of *Legionella pneumophila* by analysis of tandem repeat copy number and internal sequence variation. Microbiology 157: 2582–2594. 21622529 10.1099/mic.0.047258-0

[32] Minandri F, D’Arezzo S, Antunes LC, Pourcel C, Principe L, et al (2012) Evidence of diversity among epidemiologically related carbapenemase-producing *Acinetobacter baumannii* strains belonging to international clonal lineage II. J Clin Microbiol 50: 590–597. 22205821 10.1128/JCM.05555-11 PMC3295171

[33] Horvath P, Barrangou R (2010) CRISPR/Cas, the immune system of bacteria and archaea. Science 327: 167–170. 20056882 10.1126/science.1179555

[34] Swarts DC, Mosterd C, van Passel MW, Brouns SJ (2012) CRISPR interference directs strand specific spacer acquisition. PLoS One 7: e35888. 22558257 10.1371/journal.pone.0035888 PMC3338789

[35] Chakraborty S, Snijders AP, Chakravorty R, Ahmed M, Tarek AM, et al (2010) Comparative network clustering of direct repeats (DRs) and cas genes confirms the possibility of the horizontal transfer of CRISPR locus among bacteria. Mol Phylogenet Evol 56: 878–887. 20580935 10.1016/j.ympev.2010.05.020

[36] Grissa I, Vergnaud G, Pourcel C (2009) Clustered regularly interspaced short palindromic repeats (CRISPRs) for the genotyping of bacterial pathogens. Methods Mol Biol 551: 105–116. 19521870 10.1007/978-1-60327-999-4_9

[37] Grissa I, Vergnaud G, Pourcel C (2007) The CRISPRdb database and tools to display CRISPRs and to generate dictionaries of spacers and repeats. BMC Bioinformatics 8: 172. 17521438 10.1186/1471-2105-8-172 PMC1892036

[38] Pourcel C, Salvignol G, Vergnaud G (2005) CRISPR elements in *Yersinia pestis* acquire new repeats by preferential uptake of bacteriophage DNA, and provide additional tools for evolutionary studies. Microbiology 151: 653–663. 15758212 10.1099/mic.0.27437-0

[39] Gant VA, Wren MW, Rollins MS, Jeanes A, Hickok SS, et al (2007) Three novel highly charged copper-based biocides: safety and efficacy against healthcare-associated organisms. J Antimicrob Chemother 60: 294–299. 17567632 10.1093/jac/dkm201

[40] Falagas ME, Giannopoulou KP, Kokolakis GN, Rafailidis PI (2008) Fosfomycin: use beyond urinary tract and gastrointestinal infections. Clin Infect Dis 46: 1069–1077. 18444827 10.1086/527442

[41] Lu CL, Liu CY, Huang YT, Liao CH, Teng LJ, et al (2011) Antimicrobial susceptibilities of commonly encountered bacterial isolates to fosfomycin determined by agar dilution and disk diffusion methods. Antimicrob Agents Chemother 55: 4295–4301. 21670185 10.1128/AAC.00349-11 PMC3165352

[42] Chuang YC, Sheng WH, Li SY, Lin YC, Wang JT, et al (2011) Influence of genospecies of *Acinetobacter baumannii* complex on clinical outcomes of patients with acinetobacter bacteremia. Clin Infect Dis 52: 352–360. 21193494 10.1093/cid/ciq154

[43] Wisplinghoff H, Paulus T, Lugenheim M, Stefanik D, Higgins PG, et al (2012) Nosocomial bloodstream infections due to *Acinetobacter baumannii*, *Acinetobacter pittii* and *Acinetobacter nosocomialis* in the United States. J Infect 64: 282–290. 22209744 10.1016/j.jinf.2011.12.008

[44] Boo TW, Walsh F, Crowley B (2009) Molecular characterization of carbapenem-resistant *Acinetobacter* species in an Irish university hospital: predominance of *Acinetobacter* genomic species 3. J Med Microbiol 58: 209–216. 19141738 10.1099/jmm.0.004911-0

[45] Ko WC, Lee NY, Su SC, Dijkshoorn L, Vaneechoutte M, et al (2008) Oligonucleotide array-based identification of species in the *Acinetobacter calcoaceticus-A. baumannii* complex in isolates from blood cultures and antimicrobial susceptibility testing of the isolates. J Clin Microbiol 46: 2052–2059. 18385442 10.1128/JCM.00014-08 PMC2446839

[46] Lin YC, Sheng WH, Chang SC, Wang JT, Chen YC, et al (2008) Application of a microsphere-based array for rapid identification of *Acinetobacter* spp. with distinct antimicrobial susceptibilities. J Clin Microbiol 46: 612–617. 18039798 10.1128/JCM.01798-07 PMC2238122

[47] Lista F, Faggioni G, Valjevac S, Ciammaruconi A, Vaissaire J, et al (2006) Genotyping of *Bacillus anthracis* strains based on automated capillary 25-loci Multiple Locus Variable-Number Tandem Repeats Analysis. BMC Microbiol 6: 33. 16600037 10.1186/1471-2180-6-33 PMC1479350

[48] La Scola B, Gundi VA, Khamis A, Raoult D (2006) Sequencing of the rpoB gene and flanking spacers for molecular identification of *Acinetobacter* species. J Clin Microbiol 44: 827–832. 16517861 10.1128/JCM.44.3.827-832.2006 PMC1393131

[49] Adams MD, Goglin K, Molyneaux N, Hujer KM, Lavender H, et al (2008) Comparative genome sequence analysis of multidrug-resistant *Acinetobacter baumannii* . J Bacteriol 190: 8053–8064. 18931120 10.1128/JB.00834-08 PMC2593238

[50] Iacono M, Villa L, Fortini D, Bordoni R, Imperi F, et al (2008) Whole-genome pyrosequencing of an epidemic multidrug-resistant *Acinetobacter baumannii* strain belonging to the European clone II group. Antimicrob Agents Chemother 52: 2616–2625. 18411315 10.1128/AAC.01643-07 PMC2443898

[51] Smith MG, Gianoulis TA, Pukatzki S, Mekalanos JJ, Ornston LN, et al (2007) New insights into *Acinetobacter baumannii* pathogenesis revealed by high-density pyrosequencing and transposon mutagenesis. Genes Dev 21: 601–614. 17344419 10.1101/gad.1510307 PMC1820901

[52] Vallenet D, Nordmann P, Barbe V, Poirel L, Mangenot S, et al (2008) Comparative analysis of Acinetobacters: three genomes for three lifestyles. PLoS One 3: e1805. 18350144 10.1371/journal.pone.0001805 PMC2265553

[53] Di Nocera PP, Rocco F, Giannouli M, Triassi M, Zarrilli R (2011) Genome organization of epidemic *Acinetobacter baumannii* strains. BMC Microbiol 11: 224. 21985032 10.1186/1471-2180-11-224 PMC3224125

