# Supplementary figures and images for: Diversity of Acinetobacter baumannii in Four French Military Hospitals, as Assessed by Multiple Locus Variable Number of Tandem Repeats Analysis
Source: PLoS One. 2012 Sep 12;7(9):e44597. doi: 10.1371/journal.pone.0044597 (PMC3440325; doi:10.1371/journal.pone.0044597)

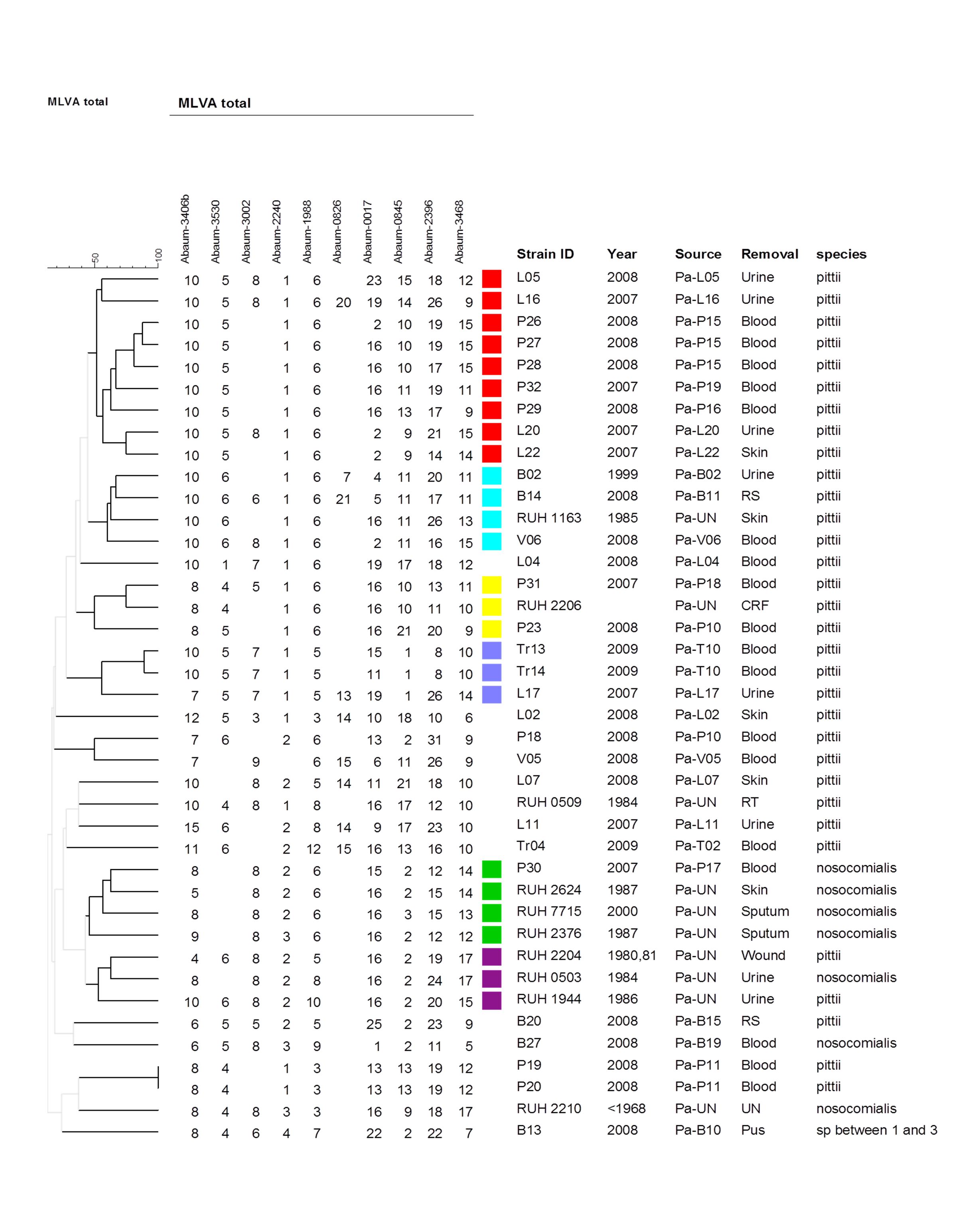

Supplement: Figure S1 — Genetic diversity of 40 non- A. baumannii ACB complex isolates. A 40% cut-off value was used to define MLVA clusters, shown with different colors when containing 3 isolates or more. On the side are indicated the year of isolation, the patient code (source), the site of isolation (removal) and the Acinetobacter species. (TIF) [file pone.0044597.s001.tif]

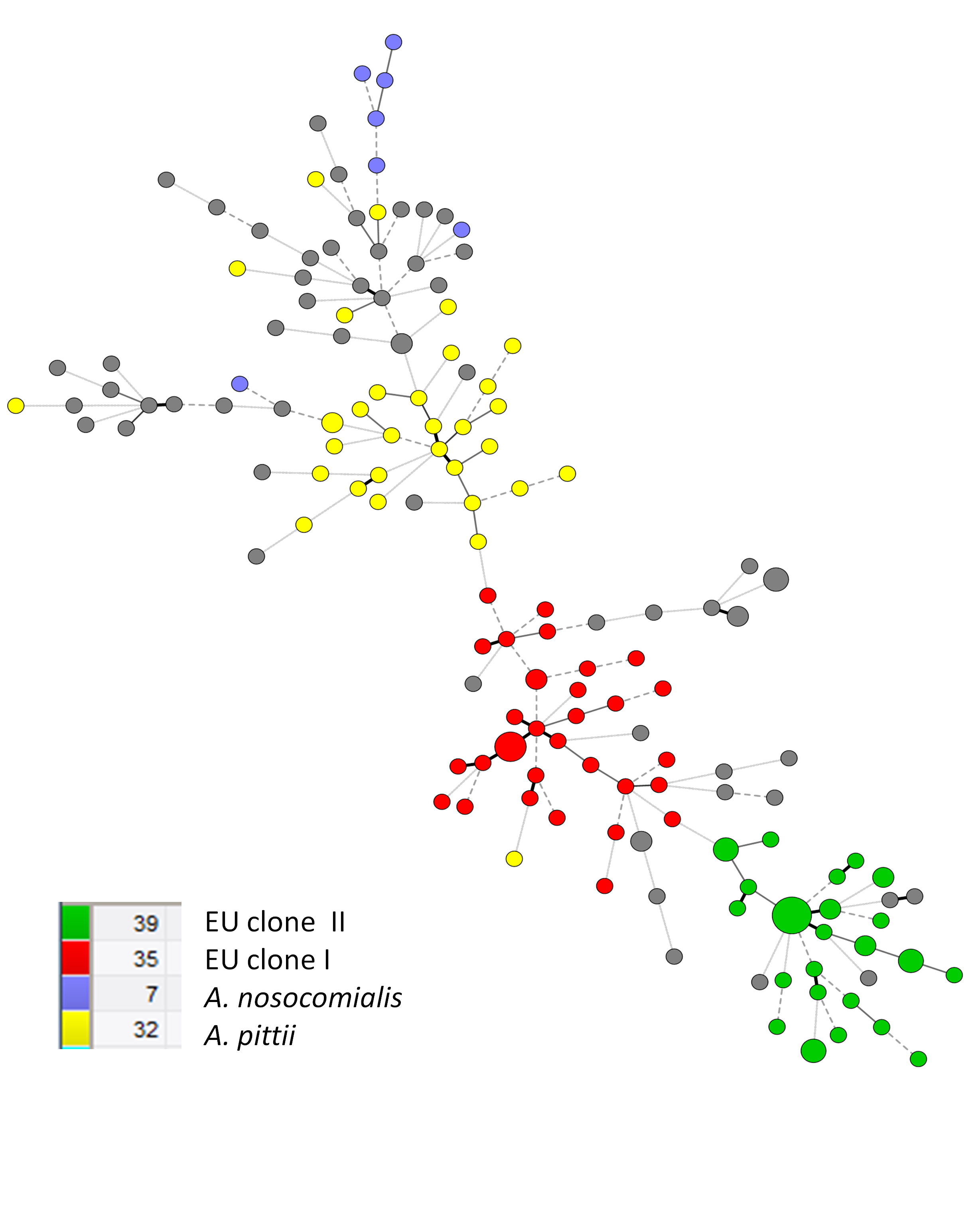

Supplement: Figure S2 — Minimum spanning analysis of MLVA data for 173 ACB complex isolates. Isolates from the present study belonging to the two larger A. baumannii clonal complexes and to the pittii and nosocomialis species are shown with colors. (TIF) [file pone.0044597.s002.tif]
