# Supplementary material for: Diversity of Acinetobacter baumannii in Four French Military Hospitals, as Assessed by Multiple Locus Variable Number of Tandem Repeats Analysis
Source: PLoS One. 2012 Sep 12;7(9):e44597. doi: 10.1371/journal.pone.0044597 (PMC3440325; doi:10.1371/journal.pone.0044597)
Supplement: Table S1 — List of isolates used in the present study. An initial is used for the different hospitals: P Percy, L Lyon, V Val de Grâce, B Béjin, Tr Trousseau. (DOC) [file pone.0044597.s004.doc]

**Table S1**

| Study code | Origin | Species | Source | Patient code | Year of isolation | Antibiotics resistance | Reference |
| --- | --- | --- | --- | --- | --- | --- | --- |
| P 01 | HIA Percy | *A. baumannii* | Respiratory tract | Pa-P01 | 2008 |  | This study |
| P 02 | HIA Percy | *A. baumannii* | Skin | Pa-P01 | 2008 |  | This study |
| P 03 | HIA Percy | *A. baumannii* | Skin | Pa-P02 | 2008 | MDR | This study |
| P 04 | HIA Percy | *A. baumannii* | Deep wound | Pa-P03 | 2008 |  | This study |
| P 05 | HIA Percy | *A. baumannii* | Skin | Pa-P01 | 2008 |  | This study |
| P 07 | HIA Percy | *A. baumannii* | Sputum | Pa-P04 | 2008 | MDR | This study |
| P 08 | HIA Percy | *E. cloacae* | Biopsy | Pa-P05 | 2008 |  | This study |
| P 09 | HIA Percy | *A. baumannii* | Respiratory tract | Pa-P06 | 2008 | MDR | This study |
| P 10 | HIA Percy | *A. baumannii* | Urine | Pa-P02 | 2008 | MDR | This study |
| P 11 | HIA Percy | *A. baumannii* | Feces | Pa-P07 | 2008 | MDR | This study |
| P 12 | HIA Percy | *A. baumannii* | Skin | Pa-P02 | 2008 | MDR | This study |
| P 13 | HIA Percy | *A. baumannii* | Skin | Pa-P02 | 2008 | MDR | This study |
| P 14 | HIA Percy | *A. baumannii* | Biopsy | Pa-P08 | 2008 |  | This study |
| P 15 | HIA Percy | *A. baumannii* | Skin | Pa-P02 | 2008 | MDR | This study |
| P 16 | HIA Percy | *A. baumannii* | Feces | Pa-P09 | 2008 | MDR | This study |
| P 17 | HIA Percy | *A. baumannii* | biop | Pa-P05 | 2008 | MDR | This study |
| P 18 | HIA Percy | *A. pittii* | Blood | Pa-P10 | 2008 |  | This study |
| P 19 | HIA Percy | *A. pittii* | Blood | Pa-P11 | 2008 |  | This study |
| P 20 | HIA Percy | *A. pittii* | Blood | Pa-P11 | 2008 |  | This study |
| P 21 | HIA Percy | 1. *ursiningii* | Blood | Pa-P12 | 2008 |  | This study |
| P 22 | HIA Percy | *A. baumannii* | Blood | Pa-P06 | 2008 | MDR | This study |
| P 23 | HIA Percy | *A. pittii* | Blood | Pa-P10 | 2008 |  | This study |
| P 24 | HIA Percy | 1. *haemolyticus* | Blood | Pa-P13 | 2008 |  | This study |
| P 25 | HIA Percy | *A. baumannii* | Blood | Pa-P14 | 2008 | MDR | This study |
| P 26 | HIA Percy | *A. pittii* | Blood | Pa-P15 | 2008 |  | This study |
| P 27 | HIA Percy | *A. pittii* | Blood | Pa-P15 | 2008 |  | This study |
| P 28 | HIA Percy | *A. pittii* | Blood | Pa-P15 | 2008 |  | This study |
| P 29 | HIA Percy | *A. pittii* | Blood | Pa-P16 | 2008 |  | This study |
| P 30 | HIA Percy | *A. pittii* | Blood | Pa-P17 | 2007 |  | This study |
| P 31 | HIA Percy | *A. pittii* | Blood | Pa-P18 | 2007 |  | This study |
| P 32 | HIA Percy | *A. pittii* | Blood | Pa-P19 | 2007 |  | This study |
| P 33 | HIA Percy | *A. baumannii* | Respiratory tract | Pa-P20 | 2009 | MDR | This study |
| P 35 | HIA Percy | *A. baumannii* | Skin | Pa-P02 | 2009 | MDR | This study |
| P 36 | HIA Percy | *A. baumannii* | Urine | Pa-P02 | 2009 | MDR | This study |
| P 37 | HIA Percy | *A. baumannii* | Skin | Pa-P20 | 2009 | MDR | This study |
| P 38 | HIA Percy | *A. baumannii* | Respiratory tract | Pa-P02 | 2009 | MDR | This study |
| P 39 | HIA Percy | *A. baumannii* | Bronchus | Pa-P02 | 2009 | MDR | This study |
| P 40 | HIA Percy | *A. baumannii* | Respiratory tract | Pa-P02 | 2009 | MDR | This study |
| P 41 | HIA Percy | *A. baumannii* | Skin | Pa-P21 | 2009 |  | This study |
| P 42 | HIA Percy | *A. baumannii* | Biopsy | Pa-P22 | 2009 |  | This study |
| P 43 | HIA Percy | *A. baumannii* | Deep wound | Pa-P25 | 2009 | XDR | This study |
| P 44 | HIA Percy | *A. baumannii* | Biopsy | Pa-P26 | 2009 | XDR | This study |
| P 45 | HIA Percy | *A. baumannii* | Skin | Pa-P27 | 2009 | XDR | This study |
| P 46 | HIA Percy | *A. baumannii* | Skin | Pa-P23 | 2009 | XDR | This study |
| P 47 | HIA Percy | *A. baumannii* | Respiratory tract | Pa-P23 | 2009 | XDR | This study |
| P 48 | HIA Percy | *A. baumannii* | Urine | Pa-P24 | 2009 | MDR | This study |
| P 49 | HIA Percy | *A. baumannii* | Skin | Pa-P23 | 2009 | MDR | This study |
| P 50 | HIA Percy | *A. baumannii* | Skin | Pa-P28 | 2009 | MDR | This study |
| P 51 | HIA Percy | *A. baumannii* | Feces | Pa-P29 | 2009 | MDR | This study |
| P 52 | HIA Percy | *A. baumannii* | Urine | Pa-P23 | 2009 | XDR | This study |
| P 53 | HIA Percy | *A. baumannii* | Skin | Pa-P24 | 2009 | MDR | This study |
| P 54 | HIA Percy | *A. baumannii* | Skin | Pa-P24 | 2009 | MDR | This study |
| P 55 | HIA Percy | *A. baumannii* | Skin | Pa-P30 | 2009 | MDR | This study |
| P 56 | HIA Percy | *A. baumannii* | Respiratory tract | Pa-P24 | 2009 | MDR | This study |
| P 57 | HIA Percy | *A. baumannii* | Skin | Pa-P23 | 2009 | MDR | This study |
| P 58 | HIA Percy | *A. baumannii* | Skin | Pa-P31 | 2010 | XDR | This study |
| P 59 | HIA Percy | *A. baumannii* | Skin | Pa-P32 | 2010 |  | This study |
| P 60 | HIA Percy | *A. baumannii* | Skin | Pa-P33 | 2010 | XDR | This study |
| P 61 | HIA Percy | *A. baumannii* | Skin | Pa-P34 | 2010 |  | This study |
| P 62 | HIA Percy | *A. baumannii* | Feces | Pa-P35 | 2010 | MDR | This study |
| P 63 | HIA Percy | *A. baumannii* | Skin | Pa-P36 | 2010 |  | This study |
| P64 | HIA Percy | *P. aeruginosa* | Skin | Pa-P37 | 2010 |  | This study |
| P 65 | HIA Percy | *A. baumannii* | Skin | Pa-P38 | 2010 | MDR | This study |
| P 66 | HIA Percy | *A. baumannii* | Deep wound | Pa-P39 | 2010 | MDR | This study |
| L 01 | HIA Desgenettes | 1. *junii* | Skin | Pa-L01 | 2008 |  | This study |
| L 02 | HIA Desgenettes | *A. pittii* | Skin | Pa-L02 | 2008 |  | This study |
| L 03 | HIA Desgenettes | *A. baumannii* | Skin | Pa-L03 | 2008 |  | This study |
| L 04 | HIA Desgenettes | *A. pittii* | Blood | Pa-L04 | 2008 |  | This study |
| L 05 | HIA Desgenettes | *A. pittii* | Urine | Pa-L05 | 2008 |  | This study |
| L 06 | HIA Desgenettes | *A. baumannii* | Urine | Pa-L06 | 2008 |  | This study |
| L 07 | HIA Desgenettes | *A. pittii* | Skin | Pa-L07 | 2008 |  | This study |
| L 08 | HIA Desgenettes | *A. baumannii* | Bronchus | Pa-L08 | 2008 |  | This study |
| L 09 | HIA Desgenettes | *A. baumannii* | Rectal swab | Pa-L09 | 2008 | MDR | This study |
| L 10 | HIA Desgenettes | *A. baumannii* | Skin | Pa-L10 | 2008 |  | This study |
| L 11 | HIA Desgenettes | *A. pittii* | Urines | Pa-L11 | 2007 |  | This study |
| L 12 | HIA Desgenettes | *A. baumannii* | Deep wound | Pa-L12 | 2007 |  | This study |
| L 13 | HIA Desgenettes | *A. baumannii* | Skin | Pa-L13 | 2007 |  | This study |
| L 14 | HIA Desgenettes | *A. baumannii* | Urine | Pa-L14 | 2007 | MDR | This study |
| L 15 | HIA Desgenettes | *A. baumannii* | Skin | Pa-L15 | 2007 |  | This study |
| L 16 | HIA Desgenettes | *A. pittii* | Urine | Pa-L16 | 2007 |  | This study |
| L 17 | HIA Desgenettes | *A. pittii* | Urine | Pa-L17 | 2007 |  | This study |
| L 18 | HIA Desgenettes | *A. baumannii* | Skin | Pa-L18 | 2007 |  | This study |
| L 19 | HIA Desgenettes | *A. baumannii* | Skin | Pa-L19 | 2007 |  | This study |
| L 20 | HIA Desgenettes | *A. pittii* | Urine | Pa-L20 | 2007 |  | This study |
| L 21 | HIA Desgenettes | *A. baumannii* | Deep wound | Pa-L21 | 2007 |  | This study |
| L 22 | HIA Desgenettes | *A. pittii* | Skin | Pa-L22 | 2007 |  | This study |
| L 23 | HIA Desgenettes | *A. baumannii* | Skin | Pa-L23 | 2007 |  | This study |
| L 24 | HIA Desgenettes | *A. baumannii* | Deep wound | Pa-L24 | 2009 | XDR | This study |
| L 25 | HIA Desgenettes | *A. baumannii* | Catheter | Pa-L25 | 2009 | XDR | This study |
| L 26 | HIA Desgenettes | *A. baumannii* | Deep wound | Pa-L24 | 2009 | XDR | This study |
| L 27 | HIA Desgenettes | *A. baumannii* | Bronchus | Pa-L24 | 2009 | XDR | This study |
| L 28 | HIA Desgenettes | *A. baumannii* | Deep wound | Pa-L24 | 2009 | XDR | This study |
| L 29 | HIA Desgenettes | *A. baumannii* | Bronchus | Pa-L25 | 2009 | XDR | This study |
| L 30 | HIA Desgenettes | *A. baumannii* | Respiratory tract | Pa-L24 | 2009 | XDR | This study |
| L 31 | HIA Desgenettes | *A. baumannii* | Catheter | Pa-L25 | 2009 | XDR | This study |
| L 32 | HIA Desgenettes | *A. baumannii* | Handle | Env-L01 | 2009 | XDR | This study |
| L 33 | HIA Desgenettes | *A. baumannii* | Dialyse | Env-L02 | 2009 | XDR | This study |
| L 34 | HIA Desgenettes | *A. baumannii* | Handle | Env-L03 | 2009 | MDR | This study |
| L 35 | HIA Desgenettes | *A. baumannii* | Respirator | Env-L01 | 2009 | XDR | This study |
| L 36 | HIA Desgenettes | *A. baumannii* | Bench | Env-L01 | 2009 | XDR | This study |
| V 01 | HIA Val De Grace | *A. baumannii* | Feces | Pa-V01 | 2009 |  | This study |
| V 02 | HIA Val De Grace | *A. baumannii* | Bronchus | Pa-V02 | 2009 | MDR | This study |
| V 03 | HIA Val De Grace | *A. baumannii* | Respiratory tract | Pa-V03 | 2007 | MDR | This study |
| V 04 | HIA Val De Grace | *A. baumannii* | Blood | Pa-V04 | 2008 |  | This study |
| V 05 | HIA Val De Grace | *A. pittii* | Blood | Pa-V05 | 2008 |  | This study |
| V 06 | HIA Val De Grace | *A. pittii* | Blood | Pa-V06 | 2008 |  | This study |
| B01 | HIA Begin | *A. baumannii* | Skin | Pa-B01 | 1999 |  | This study |
| B02 | HIA Begin | *A. pittii* | Urine | Pa-B02 | 1999 |  | This study |
| B04 | HIA Begin | *A. baumannii* | Urine | Pa-B04 | 2000 |  | This study |
| B05 | HIA Begin | *A. baumannii* | Rectal swab | Pa-B05 | 2000 |  | This study |
| B08 | HIA Begin | *A. baumannii* | Unknown | Pa-B06 | 2000 |  | This study |
| B09 | HIA Begin | *A. baumannii* | Unknown | Pa-B07 | 2000 |  | This study |
| B10 | HIA Begin | *A. baumannii* | Unknown | Pa-B08 | 1999 |  | This study |
| B11 | HIA Begin | *A. baumannii* | Skin | Pa-B09 | 2008 |  | This study |
| B13 | HIA Begin | 1. *sp between 1 and 3* | Skin | Pa-B10 | 2008 |  | This study |
| B14 | HIA Begin | *A. pittii* | Rectal swab | Pa-B11 | 2008 |  | This study |
| B15 | HIA Begin | *A. baumannii* | catheter | Pa-B12 | 2008 |  | This study |
| B16 | HIA Begin | *A. baumannii* | Skin | Pa-B13 | 2007 |  | This study |
| B17 | HIA Begin | *A. baumannii* | Respiratory tract | Pa-B13 | 2007 |  | This study |
| B18 | HIA Begin | *A. baumannii* | catheter | Pa-B13 | 2007 |  | This study |
| B19 | HIA Begin | *A. baumannii* | Respiratory tract | Pa-B14 | 2007 | MDR | This study |
| B20 | HIA Begin | *A. pittii* | Rectal swab | Pa-B15 | 2008 | MDR | This study |
| B21 | HIA Begin | *A. baumannii* | Rectal swab | Pa-B16 | 2008 |  | This study |
| B22 | HIA Begin | *A. baumannii* | Skin | Pa-B17 | 2007 |  | This study |
| B23 | HIA Begin | *A. baumannii* | Respiratory tract | Pa-B18 | 2008 |  | This study |
| B24 | HIA Begin | *A. baumannii* | sinus | Pa-B18 | 2008 |  | This study |
| B25 | HIA Begin | *A. baumannii* | Rectal swab | Pa-B18 | 2008 |  | This study |
| B27 | HIA Begin | 1. *nosocomialis* | Blood | Pa-B19 | 2008 |  | This study |
| B28 | HIA Begin | *A. baumannii* | catheter | Pa-B20 | 2007 |  | This study |
| Tr01 | APHP Trousseau | 1. *lwofii* | Blood | Pa-T01 | 2009 |  | This study |
| Tr02 | APHP Trousseau | *A. baumannii* | Blood | Pa-T02 | 2009 | MDR | This study |
| Tr03 | APHP Trousseau | *A. baumannii* | Respiratory tract | Pa-T02 | 2009 | MDR | This study |
| Tr04 | APHP Trousseau | *A. pittii* | Blood | Pa-T03 | 2009 |  | This study |
| Tr05 | APHP Trousseau | 1. *lwofii* | Blood | Pa-T04 | 2009 |  | This study |
| Tr06 | APHP Trousseau | 1. *lwofii* | Blood | Pa-T05 | 2009 |  | This study |
| Tr07 | APHP Trousseau | *A. baumannii* | Pus | Pa-T06 | 2009 |  | This study |
| Tr08 | APHP Trousseau | *A. baumannii* | Pus | Pa-T07 | 2009 |  | This study |
| Tr09 | APHP Trousseau | *A. baumannii* | Blood | Pa-T08 | 2009 | MDR | This study |
| Tr10 | APHP Trousseau | 1. *lwofii* | Blood | Pa-T09 | 2009 |  | This study |
| Tr11 | APHP Trousseau | *spp* | Blood | Pa-T09 | 2009 |  | This study |
| Tr12 | APHP Trousseau | *spp* | Blood | Pa-T09 | 2009 |  | This study |
| Tr13 | APHP Trousseau | *A. pittii* | Blood | Pa-T10 | 2009 |  | This study |
| Tr14 | APHP Trousseau | *A. pittii* | Blood | Pa-T10 | 2009 |  | This study |
| Tr15 | APHP Trousseau | *A. baumannii* | Pus | Pa-T11 | 2009 | XDR | This study |
| Tr16 | APHP Trousseau | *A. baumannii* | Biopsy | Pa-T11 | 2009 | XDR | This study |
| Tr17 | APHP Trousseau | *A. baumannii* | Blood | Pa-T12 | 2009 |  | This study |
| Tr18 | APHP Trousseau | 1. *lwofii* | Blood | Pa-T13 | 2009 |  | This study |
| Tr19 | APHP Trousseau | *A. baumannii* | Blood | Pa-T14 | 2009 |  | This study |
| Tr20 | APHP Trousseau | *spp* | Blood | Pa-T15 | 2009 |  | This study |
| Tr21 | APHP Trousseau | *spp* | Blood | Pa-T15 | 2009 |  | This study |
| Tr22 | APHP Trousseau | 1. *lwofii* | Blood | Pa-T16 | 2009 |  | This study |
| ACICU | Italy | *A. baumannii* | CF | Pa-UN | ACICU |  |  |
| AYE | France | *A. baumannii* | Urine | Pa-UN | AYE |  |  |
| SDF | France | *A. baumannii* | body louse | Pa-UN | SDF |  |  |
| RUH 134 | Netherlands | *A. baumannii* | Urine | Pa-UN | 1982 |  |  |
| RUH 875 | Netherlands | *A. baumannii* | Urine | Pa-UN | 1984 |  |  |
| RUH 5875 | Netherlands | *A. baumannii* | Unknown | Pa-UN | 1997 |  |  |
| RUH 0509 | Netherlands | *A. pittii* | Bronchus | Pa-UN | 1984 |  |  |
| RUH 1163 | Netherlands | *A. pittii* | Toe web | Pa-UN | 1985 |  |  |
| RUH 1944 | Netherlands | *A. pittii* | Urine | Pa-UN | 1986 |  |  |
| RUH 2204 | Sweden | *A. pittii* | Wound | Pa-UN | 1980,81 |  |  |
| RUH 2206 | Unknown | *A. pittii* | Cerebrospinal fluid | Pa-UN | Unknown |  |  |
| RUH 0503 | Netherlands | 1. *nosocomialis* | Urine | Pa-UN | 1984 |  |  |
| RUH 2210 | Unknown | 1. *nosocomialis* | Unknown | Pa-UN | <1968 |  |  |
| RUH 2376 | Netherlands | 1. *nosocomialis* | Sputum | Pa-UN | 1987 |  |  |
| RUH 2624 | Netherlands | 1. *nosocomialis* | Skin | Pa-UN | 1987 |  |  |
| RUH 7715 | Netherlands | 1. *nosocomialis* | Sputum | Pa-UN | 2000 |  |  |
| ATCC 17978 | [NC_009085](http://www.ncbi.nlm.nih.gov.gate1.inist.fr/nucleotide/126640115/) | *A. baumannii* | Cerebrospinal fluid | Pa-UN | 1951 |  |  |
| AB0057 | [NC_011586](http://www.ncbi.nlm.nih.gov.gate1.inist.fr/nucleotide/213155370/) | *A. baumannii* | Blood | Pa-UN | 2004 |  |  |
| AB307-0294 | [CP001172](http://www.ncbi.nlm.nih.gov/nucleotide/213985689/) | *A. baumannii* | Blood | Pa-UN | 1994 |  |  |

1. **Adams, M. D., K. Goglin, N. Molyneaux, K. M. Hujer, H. Lavender, J. J. Jamison, I. J. MacDonald, K. M. Martin, T. Russo, A. A. Campagnari, A. M. Hujer, R. A. Bonomo, and S. R. Gill.** 2008. Comparative genome sequence analysis of multidrug-resistant *Acinetobacter baumannii*. J Bacteriol **190:**8053-64.

2. **Dijkshoorn, L., R. van Dalen, A. van Ooyen, D. Bijl, I. Tjernberg, M. F. Michel, and A. M. Horrevorts.** 1993. Endemic acinetobacter in intensive care units: epidemiology and clinical impact. J Clin Pathol **46:**533-6.

3. **Iacono, M., L. Villa, D. Fortini, R. Bordoni, F. Imperi, R. J. Bonnal, T. Sicheritz-Ponten, G. De Bellis, P. Visca, A. Cassone, and A. Carattoli.** 2008. Whole-genome pyrosequencing of an epidemic multidrug-resistant *Acinetobacter baumannii* strain belonging to the European clone II group. Antimicrob Agents Chemother **52:**2616-25.

4. **Janssen, P., K. Maquelin, R. Coopman, I. Tjernberg, P. Bouvet, K. Kersters, and L. Dijkshoorn.** 1997. Discrimination of *Acinetobacter* genomic species by AFLP fingerprinting. Int J Syst Bacteriol **47:**1179-87.

5. **Nemec, A., L. Dijkshoorn, and T. J. van der Reijden.** 2004. Long-term predominance of two pan-European clones among multi-resistant *Acinetobacter baumannii* strains in the Czech Republic. J Med Microbiol **53:**147-53.

6. **Smith, M. G., T. A. Gianoulis, S. Pukatzki, J. J. Mekalanos, L. N. Ornston, M. Gerstein, and M. Snyder.** 2007. New insights into *Acinetobacter baumannii* pathogenesis revealed by high-density pyrosequencing and transposon mutagenesis. Genes Dev **21:**601-14.

7. **Vallenet, D., P. Nordmann, V. Barbe, L. Poirel, S. Mangenot, E. Bataille, C. Dossat, S. Gas, A. Kreimeyer, P. Lenoble, S. Oztas, J. Poulain, B. Segurens, C. Robert, C. Abergel, J. M. Claverie, D. Raoult, C. Medigue, J. Weissenbach, and S. Cruveiller.** 2008. Comparative analysis of Acinetobacters: three genomes for three lifestyles. PLoS One **3:**e1805.

8. **van Dessel, H., L. Dijkshoorn, T. van der Reijden, N. Bakker, A. Paauw, P. van den Broek, J. Verhoef, and S. Brisse.** 2004. Identification of a new geographically widespread multiresistant *Acinetobacter baumannii* clone from European hospitals. Res Microbiol **155:**105-12.

9. **van Dessel, H., T. E. Kamp-Hopmans, A. C. Fluit, S. Brisse, A. M. de Smet, L. Dijkshoorn, A. Troelstra, J. Verhoef, and E. M. Mascini.** 2002. Outbreak of a susceptible strain of *Acinetobacter* species 13 (sensu Tjernberg and Ursing) in an adult neurosurgical intensive care unit. J Hosp Infect **51:**89-95.
