# Supplementary material for: Diversity of Acinetobacter baumannii in Four French Military Hospitals, as Assessed by Multiple Locus Variable Number of Tandem Repeats Analysis
Source: PLoS One. 2012 Sep 12;7(9):e44597. doi: 10.1371/journal.pone.0044597 (PMC3440325; doi:10.1371/journal.pone.0044597)
Supplement: Table S2 — Result of MLVA genotyping of reference strains. (DOCX) [file pone.0044597.s005.docx]

**Table S2**

| Strain ID | Acinetobacter | VNTR repeats number | | | | | | | | | |
| --- | --- | --- | --- | --- | --- | --- | --- | --- | --- | --- | --- |
|  | species | Abaum-3406 | Abaum-3530 | Abaum-3002 | Abaum-2240 | Abaum-1988 | Abaum-0826 | Abaum-0017 | Abaum-0845 | Abaum-2396 | Abaum-3468 |
| ACICU | *baumannii* | 0 | 7 | 7 | 4 | 9 | 13 | 14 | 10 | 20 | 13 |
| ATCC17978 | *baumannii* | 10 | 6 | 8 | 2 | 5 | 19 | 18 | 2 | 19 | 13 |
| AYE | *baumannii* | 15 | 6 | 7 | 3 | 6 | 17 | 11 | 19 | 21 | 14 |
| RUH134 | *baumannii* | 0 | 7 | 7 | 4 | 9 | 15 | 12 | 8 | 20 | 12 |
| RUH875 | *baumannii* | 15 | 6 | 7 | 3 | 6 | 15 | 20 | 14 | 20 | 16 |
| RUH5875 | *baumannii* | 8 | 6 | 8 | 4 | 5 | 6 | 9 | 0 | 10 | 13 |
| RUH 0509 | *pittii* | 10 | 4 | 8 | 1 | 8 | 0 | 16 | 17 | 12 | 10 |
| RUH 1163 | *pittii* | 10 | 6 | 0 | 1 | 6 | 0 | 16 | 11 | 26 | 13 |
| RUH 1944 | *pittii* | 10 | 6 | 8 | 2 | 10 | 0 | 16 | 2 | 20 | 15 |
| RUH 2204 | *pittii* | 4 | 6 | 8 | 2 | 5 | 0 | 16 | 2 | 19 | 17 |
| RUH 2206 | *pittii* | 8 | 4 | 0 | 1 | 6 | 0 | 16 | 10 | 11 | 10 |
| RUH 0503 | *nosocomialis* | 8 | 0 | 8 | 2 | 8 | 0 | 16 | 2 | 24 | 17 |
| RUH 2210 | *nosocomialis* | 8 | 4 | 8 | 3 | 3 | 0 | 16 | 9 | 18 | 17 |
| RUH 2376 | *nosocomialis* | 9 | 0 | 8 | 3 | 6 | 0 | 16 | 2 | 12 | 12 |
| RUH 2624 | *nosocomialis* | 5 | 0 | 8 | 2 | 6 | 0 | 16 | 2 | 15 | 14 |
| RUH 7715 | *nosocomialis* | 8 | 0 | 8 | 2 | 6 | 0 | 16 | 3 | 15 | 13 |
