# Supplementary material for: Diversity of Acinetobacter baumannii in Four French Military Hospitals, as Assessed by Multiple Locus Variable Number of Tandem Repeats Analysis
Source: PLoS One. 2012 Sep 12;7(9):e44597. doi: 10.1371/journal.pone.0044597 (PMC3440325; doi:10.1371/journal.pone.0044597)
Supplement: Table S3 — List of spacers identified in sequenced AYE CRISPR loci. 1 The table corresponds to the dictionary produced by CRISPRcompar [Grissa, 2008 #893] where spacers are annotated in each analysed sample as shown in the second column. (DOCX) [file pone.0044597.s006.docx]

**Table S3**

| **key** | **Annotated Spacer^1^** | **sequence** |
| --- | --- | --- |
| 1 | AYE:1_AB0057:1_AB307:1 | ATCACATCACTTCATCTCCTCAACCTGAACGC |
| 2 | AYE:2_AB0057:2_AB307:2 | GTAAAAGTGACTTACCACAACCGTGGTTATTT |
| 3 | AYE:3_AB0057:3_AB307:3 | CTTATGCTTTCTTCCGTTATCCGTACGCCAAC |
| 4 | AYE:4_AB0057:4_AB307:4 | AAGTATTTTAAAGCATAAATCCTTTTAGCAAA |
| 5 | AYE:5_AB0057:5_AB307:5 | AAATGACTGTGACGGGAGGTGGATCGCCTTGA |
| 6 | AYE:6_AB0057:6_AB307:6 | GCGGTATATTGTTTTATTCTTTACTTGGAGAA |
| 7 | AYE:7_AB0057:7_AB307:7 | TTATCCCCTACGCACTTGAAAAGATTTCCGCT |
| 8 | AYE:8_AB0057:8_AB307:8 | ATTTCGGGCACTTATGAATAACTCGTTCAACA |
| 9 | AYE:9_AB0057:9_AB307:9 | TGTTGGATATTGTCCAATTTCCTCATTTTTTGG |
| 10 | AYE:10_AB0057:10_AB307:10 | TTATCGGGGTTGTGAATGGCCGGCCAATGACA |
| 11 | AYE:11_AB0057:11_AB307:11 | AAATTTTTACCCCCATAAATAGCTTTTAGTTG |
| 12 | AYE:12_AB0057:12_AB307:12 | TATTTTTCTAACTTTGGTTCAAGTCGTGTTCG |
| 13 | AYE:13_AB0057:13_AB307:13 | CTCCCCACATCTTGACCAAATTGCACATCCAA |
| 14 | AYE:14_AB0057:14_AB307:14 | TCTCGTCTTTCTCAGTCATAATCTTTGTCCAA |
| 17 | AYE:15_AB0057:15_AB307:17 | TATTCGTGACCTTGTCCAAATCTGCGAGATAT |
| 18 | AYE:16_AB0057:16_AB307:18 | TTTTTGGCATCTCAAGAACTTGCTCGTGCTAT |
| 19 | AYE:17_AB0057:17_AB307:19 | ACAAAAACTCATGCCATCGTAACTGATAAAGAC |
| 20 | AYE:18_AB0057:18_AB307:20 | AAATAGTTCCCATAATACTTGCGTAGGTTGAA |
| 21 | AYE:19_AB0057:19_AB307:21 | CATATGTCACTTCACCATCTGAATTTCGGACA |
| 22 | AYE:20_AB0057:20_AB307:22 | AAGGCATATTTGCAAAACCTTCACGATGAGAA |
| 23 | AYE:21_AB0057:21 | TTTGCTTTGATGTTTCATTCCCAGCAATAAAA |
| 24 | AYE:22_AB0057:22 | TTGCGACTTTGATTTCTACTTTAGCTATCAAA |
| 25 | AYE:23_AB0057:23 | TACTGGTGGCTCATACCACTCAGGCAATTGTT |
| 26 | AYE:24_AB0057:24 | GTTACATCGATACGGGTCATATTGTTGTACAA |
| 27 | AYE:25_AB0057:25 | ACGTACTCAGCCAAATTGTCGGATCAGACAAC |
| 28 | AYE:26_AB0057:26 | TCTTAGTGAATCACCGCCACTCTCTACTTTTA |
| 29 | AYE:27_AB0057:27 | CGGAAGGGTATTTTAGAGTTAACAGAAAAAAT |
| 30 | AYE:28_AB0057:28 | CCTGCCCCGAACAATATGATGCTTTTTTTCAA |
| 31 | AYE:29_AB0057:29 | GATTTAAGAAAGTTTATAAGCAGTTTTAATAC |
| 32 | AYE:30_AB0057:30_AB307:23 | ACTGGTACCCGATTTTCAACGTGGGCATGTAT |
| 33 | AYE:31_AB0057:31_AB307:24 | TTTTAATGCACATGAAGGCCACTAATACAGAA |
| 34 | AYE:32_AB0057:32_AB307:25 | CTGAACAGGTAACTGCTGATCTACTTGATGCT |
| 35 | AYE:33_AB0057:33_AB307:26 | AACTTGCTCAATGATTTACGAAATCCAATTCA |
| 36 | AYE:34_AB0057:34_AB307:27 | AATTTTATGTAACTGCTTTTAAATCCCTTTCA |
| 37 | AYE:35_AB0057:35_AB307:28 | GATAACCCAAGCCAAGCCTATAAGTTTCAAAA |
| 38 | AYE:36_AB0057:36_AB307:29 | CCTGTGACAACTGGTGAGTTGGTCACGCTTGG |
| 39 | AYE:37_AB0057:37_AB307:30 | CCAATGGGCAACTTTGGAAACGCTACGCCACA |
| 40 | AYE:38_AB307:31 | GATTCTCAACAACTATCAGTGGTCTTGCAGAT |
| 41 | AYE:39_AB0057:38_AB307:32 | CCTTCACTCACAGCGGGAGTAAATGCCTATGC |
| 42 | AYE:40_AB0057:39_AB307:33 | ATTTCAGGCAATAAAAAACCCCGCTTTCGCAG |
| 43 | AYE:41_AB0057:40_AB307:34 | AAATACGATATCTATAAGGGCTATGATCATGA |
| 44 | AYE:42_AB0057:41_AB307:35 | TGGAAGTCATAGACCGTATGGTTGCGGCCCAT |
| 45 | AYE:43_AB0057:42_AB307:36 | AGATAGAAAAGACTAAAAGCAATGATGTAACT |
| 46 | AYE:44_AB0057:43_AB307:37 | AGATGCTGAGCAATTTCCTATTGTTGCATATT |
| 47 | AYE:45_AB0057:44_AB307:38 | TGATATTCACACATAATTTTTATGCTTATGCT |
| 48 | AYE:46_AB0057:45_AB307:39 | ACAAATTTTCTTGTAGCTGTATTGATGAGCAA |
| 49 | AYE:47_AB0057:46_AB307:40 | TCAACAACAAGATATTGAACAAGACTTTGGAA |
| 50 | AYE:48_AB0057:47_AB307:41 | CAAAACAACTTAAAGGCACCAGTTACCCCAAA |
| 51 | AYE:49_AB0057:48_AB307:42_A28:1 | CACCCGTAGTTGAATCAACACGTAAAATATAT |
| 52 | AYE:50_AB0057:49_AB307:43_A28:2_P54:1_P65:1 | ATTCTTGTAGTGAGATTGAATTGTATTACCAT |
| 53 | AYE:51_AB0057:50_AB307:44_A28:3_P54:2_P65:2 | GTATGCAAATCTTGGAGGCTTAACCGCTTTGG |
| 54 | AYE:52_AB0057:51_AB307:45_A28:4_P54:3_P65:3 | TATCCAATTACACGTTCAAAAACAGCGCAACA |
| 55 | AYE:53_AB0057:52_P65:4 | CCAAAGGCGCTTACCCTCAAAGCCTTTGATGA |
| 56 | AYE:54 | CGATTAATTTAGATGGTACTGTCACTTACAAC |
| 57 | AYE:55 | ACAAAAAGCCCGTAGGTGGTCGATCTGTAACA |
| 58 | AYE:56 | CTATCAAGGCGCTGCTGATCAATTCCTTGTCT |
| 59 | AYE:57 | CTAAAAATGTTTTAAGTGCACTTAAAGTAGCT |
| 60 | AYE:58 | GCGTTAAAGCGCAAAACCTTGTTGATTATATC |
| 61 | AYE:59 | AATTAAAAACCCACCTGATTAATGCCCAACCA |
| 15 | AB307:15 | ATGATAAGGCATCGCCTGAGCATTACATTGAT |
| 16 | AB307:16 | CTTTTAAAGCGAAATCTGGTTACACAGTTGAA |
| 62 | A28:5 | CAATAGGGGTAGTAGAACGTATTCTTCTTAGT |
| 63 | A28:6 | TATTGTTTGATCTTGGGGTGGTTCTGCATTTG |
| 64 | A28:7 | ATACAGGGATTAAGACATTCACTGTTCAAGAT |
| 65 | P54:4 | AGGAGGACTTGACCACGTTCAAGTCTTTCAAA |
| 66 | P54:5 | AGCATGTTGTTGGATGTTGAATCGGGAGCTGA |
| 67 | P54:6 | GGGCGTATCGGGTAAGGGTTTTATCGGCGGAT |
| 68 | P54:7 | TTTAGATCGTGCATCTGCAAGACTTACTTCTG |
| 69 | P54:8 | TATGCTTCAATGGCACGCTTGAACGCGCCACT |
| 70 | P54:9 | ATGATCACCTCAATAAAAAACCACCCGAGGGT |
| 71 | P54:10 | GTACAAAAACGGCAAAGGGACGGATAGTCCTT |
| 72 | P54:11 | TTTTCAAGACCGCATCCATTAGGCCGAAACTT |
| 73 | P54:12 | ATGATCACCCCAAAAGAAAACCTCCCGAAGGA |
| 74 | P54:13 | GTTTACTTGCTGGCGGCAATAAGTATGCACAA |
| 75 | P54:14 | AGCATGTTTAACCCTCCATAGAATTTGGCGAAG |
| 76 | P54:15 | TTTCAAAGACGTGGCTGGTTGAAGCGTTTGAA |
| 77 | P54:16 | AGGCCAAACAGGAAGGCCAACTTCTATTTTGAT |
| 78 | P54:17 | CTGGGTAATGTCGAATATCTCTAACATTCCCAATT |
| 79 | P54:18 | TCGTCAATACGTTCTAAAGCATTCTTCTGCATC |
| 80 | P65:5_P65:6_P65:9 | ATTTCCCAATTGTGTGAGTGTGGCTTTTCCAA |
| 81 | P65:7 | TTTTTCTTCATTTTCTTGTGGTGGTGCTTTCG |
| 82 | P65:8 | GTTACTACCAGGTATGAACTAGGACTTTACCA |
| 83 | P65:10 | TTATCTGAACTTTGCGAATGTGGCTTTTCAAA |
| 84 | P65:11 | ATTAACAAAGATGCTTGATTGGTCTCGTGCTT |
| 85 | P65:12 | TTAAACAAGTTGAAGAAGAAGTAAAAAAAAGC |
| 86 | P65:13 | CAGTTATGAAAAAAGCTGGGTGCGGTGCGTAC |
| 87 | P65:14 | AAAGAGGATTTTGATCTTTTGAACATCTCAGT |
| 88 | P65:15 | TTTTAACTTCTTCAATTTGCATATGCCTTA |
| 89 | P65:16 | CAGACATGATTTGAGAGCTACGGCATCATAC |
| 90 | P65:17 | TATCTGAATAGTCAGAATGATAGTGACTCATACT |
| 91 | P65:18 | CTTGCCCAAGCACCTGACTGAAAATACTTTATTA |
| 92 | P65:19 | AGGATTCAGTTATCGCTAAGCTGCATAGGCTTT |
| 93 | P65:20 | AAAAAAGATTAATTAAATATTCAATTTGCGC |
| 94 | P65:21 | ATAACGATCTGTAAATGTAATGGATGCTAAAC |
| 95 | P65:22 | TTTTTAACTCAGTTGCAATGATTTTTGGGAAT |
| 96 | P65:23 | ATATGGTTCTTTCATTAGAACCTAGAGTATGT |
| 97 | P65:24 | TAACGAACATTATGCAACCCATCTCTTGAGAA |
| 98 | P65:25 | TGGCTTTTCACATGTCTAAATGTGAAGGGTAA |
| 99 | P65:26 | TTTAATCGAGATCAGCTTTTTTGCTTCACTTC |
| 100 | P65:27 | ATAATCAGGTAAATGAGATTGCAGAGGGCGTA |
| 101 | P65:28 | TTTTAGTTGACTTGATGTAAATTTCACGTAAC |
| 102 | P65:29 | ACATTTAAAGACATAAGTCGGGCGTTGAGTTG |
| 103 | P65:30 | GATTACTGACGCATGATGCTTTTTTTTGGTAT |
| 104 | P65:31 | AAAAGACAGTTTTTCACGGGTAATATTTGCAG |
| 105 | P65:32 | GTTTTGTTGTTCCAGTTCTTCATGCTGTTGTT |
| 106 | P65:33 | TTTGTACTACTTTATCTCTTTTATCCTTTGCAG |
| 107 | P65:34 | GAAAAATCCCCATCTAATTTAAAATCAGATGA |
| 108 | P65:35 | TATAAAATTATAGTAAAAATATAAGTACAA |
